# Dodecanedioic acid prevents and reverses metabolic‐associated liver disease and obesity and ameliorates liver fibrosis in a rodent model of diet‐induced obesity

**DOI:** 10.1096/fj.202402108R

**Published:** 2024-11-26

**Authors:** Giulia Angelini, Sara Russo, Fabrizia Carli, Patrizia Infelise, Simona Panunzi, Alessandro Bertuzzi, Maria Emiliana Caristo, Erminia Lembo, Roberta Calce, Stefan R. Bornstein, Amalia Gastaldelli, Geltrude Mingrone

**Affiliations:** ^1^ Department of Translational Medicine and Surgery Università Cattolica del Sacro Cuore Rome Italy; ^2^ Department of Medical and Surgical Sciences Fondazione Policlinico Universitario A. Gemelli IRCCS Rome Italy; ^3^ Cardiometabolic Risk Laboratory Institute of Clinical Physiology (IFC), National Research Council (CNR) Pisa Italy; ^4^ CNR‐IASI, Laboratorio di Biomatematica, Consiglio Nazionale delle Ricerche Istituto di Analisi dei Sistemi ed Informatica Rome Italy; ^5^ CNR‐IASI, Consiglio Nazionale delle Ricerche Istituto di Analisi dei Sistemi ed Informatica, Laboratorio di Biomatematica Rome Italy; ^6^ Department of Medicine III Universitätsklinikum Carl Gustav Carus an der Technischen Universität Dresden Dresden Germany; ^7^ Division of Diabetes & Nutritional Sciences, School of Cardiovascular and Metabolic Medicine & Sciences King's College London London UK

**Keywords:** citrate, de novo lipogenesis, dodecanedioic acid (DC12), glucose tolerance, metabolic dysfunction‐associated steatohepatitis (MASH), obesity

## Abstract

Dodecanedioic acid (DC12) is a dicarboxylic acid present in protective polymers of fruit and leaves. We explored the effects of DC12 on metabolic dysfunction‐associated steatohepatitis (MASH) and obesity. DC12 supplementation (100 mg/kg/day) was added to a high‐fat diet (HFD) for 8 weeks in rodents to assess its impact on obesity and MASH prevention. Rats given DC12 experienced significant reductions of weight gain, liver and visceral fat weight, and improved glucose tolerance and insulin sensitivity. Liver histology showed protection against diet‐induced MASH, with reduced steatosis, hepatocyte ballooning, and fibrosis. For weight‐loss and MASH reversion, rats were fed HFD for 14 weeks, followed by 6 weeks with or without DC12. DC12 supplementation (100 mg/kg/day) led to a significant reduction of weight gain and liver weight. DC12 induced white adipose tissue beiging and reduced adiposity with a decrease of visceral fat. It also improved glucose tolerance, insulin sensitivity, and reduced hepatic gluconeogenic gene expression. Liver histology revealed a significant reduction in steatosis, hepatocyte ballooning, and inflammation as well as fibrosis, indicating MASH reversal. DC12 reduced hepatic lipogenesis enzymes as well as de novo lipogenesis measured by deuterated water and increased fatty acid β‐oxidation. Plasma lipid profile showed lower triglycerides and phosphatidylcholines in the DC12 group. Notably, DC12 decreased mINDY expression, the cell membrane Na+‐coupled citrate transporter, reducing citrate uptake and de‐novo lipogenesis, linking its effects to improved lipid metabolism and reduced steatosis. We found that during the hepatic first pass, half of the DC12 ingested with water was taken up by the liver. The concentration of DC12 in the portal vein falls within the range identified in vitro as sufficient to inhibit citrate transport in hepatocytes.

## Graphical abstract

Dodecanedioic acid (DC12) demonstrates a protective and therapeutic effect against metabolic‐associated steatohepatitis (MASH) and obesity by targeting key metabolic pathways in the liver. DC12 modulates multiple aspects of hepatic lipid metabolism, including the inhibition of de novo lipogenesis and enhancement of fatty acid β‐oxidation. DC12 reduces hepatic steatosis, inflammation, and fibrosis ‐hallmarks of MASH progression‐ highlighting its potential as a promising option for both the prevention and treatment of MASH.

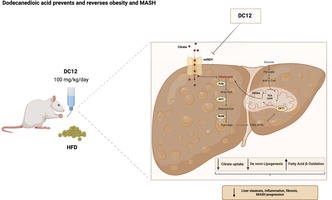

**Abbreviations**
ACC1: acetyl‐CoA carboxylase 1
ACLY: ATP‐citrate lyase
CPT1: carnitine palmitoyltransferase 1
CVD: cardiovascular disease
DC12: dodecanedioic acid
DGAT: diacylglycerol acyltransferase
DNL: de novo lipogenesis
FASN: fatty acid synthase
HFD: high‐fat diet
HOMA‐IR: homeostatic model assessment for insulin resistance
MASH: metabolic dysfunction‐associated steatohepatitis
NaCT: sodium‐coupled citrate transporter
Pgc1a: PPARG coactivator 1 alpha
Prdm16: PR/SET domain 16
SAT: subcutaneous adipose tissue
Ucp1: Uncoupling protein 1
VLDL: very low‐density lipoproteins

## 1 INTRODUCTION

Dodecanedioic acid (DC12) is a medium‐chain aliphatic α,ω‐dicarboxylic acid that is present in nature as a component of cutin and suberin.^1^ Cutin is a waxy polymer composed primarily of hydroxy and hydroxyepoxy fatty acids and functions as a critical component of the plant cuticle, providing a barrier that minimizes water loss and protects against pathogens. Suberin is present in the inner cell wand next to the plasma membrane at the level of root endodermal and exodermal cell layers.

We extensively studied DC12 use in both animals and humans, primarily for its potential metabolic and therapeutic benefits.^2^, ^3^, ^4^, ^5^ As an energy source, DC12 can be metabolized via the Krebs' cycle, providing a steady source of energy without significantly raising blood glucose or insulin levels.^6^ This property makes dicarboxylic acids particularly interesting for use in metabolic disorders such as diabetes.^7^ Research in people with type 2 diabetes suggests that DC12 supplementation may improve muscle function and endurance and overcome metabolic inflexibility.^8^ Its slow and sustained oxidation process offers a prolonged energy release, which is beneficial during extended physical activities.^8^

In clinical settings, DC12 has been explored as a component of specialized diets for patients with specific metabolic needs.^2^, ^3^, ^4^, ^5^, ^6^, ^8^ Research on the safety profile of DC12 suggests that it is generally well‐tolerated in both animals and humans.^2^, ^3^, ^4^, ^5^

We hypothesized that DC12 could improve liver function, which is counteracting the effects of a high‐fat diet (HFD) that causes metabolic dysfunction‐associated steatohepatitis (MASH) in rodents by inhibiting the citrate/Na^+^ symporter, highly active in hepatocytes. This transporter facilitates the cotransport of various di‐ and tri‐carboxylic acids.^9^

The gene product of Slc13a5 encodes the sodium‐coupled citrate transporter (NaCT), also referred to as mINDY.^9^ This transporter is integral to cellular metabolism as it facilitates the uptake of citrate from the bloodstream into cells by coupling the transport process to the movement of sodium ions. Citrate, a key intermediate in the citric acid cycle, plays a crucial role in de novo lipogenesis (DNL).^10^ In fact, mINDY‐KO mice show reduced hepatic lipogenesis, enhanced hepatic lipid oxidation, and increased insulin sensitivity.^11^

In order to study the effect of DC12 in the prevention of MASH as well as in the improvement of established MASH, we have administered 100 mg/kg/day of DC12 as a sodium salt in drinking water along with a HFD diet for 8 weeks or 100 mg/kg/day of DC12 sodium salt in drinking water after 14 weeks of a HFD diet that continued in association with DC12 for a further 6 weeks.

## 2 MATERIALS AND METHODS

### 2.1 Study design

Forty adult Wistar male rats, aged 8–10 weeks, were included in the study. The rats were housed in individual cages at 22°C with 12‐h light cycles and had ad libitum access to food and water. Following one week of acclimation, rats were randomly divided into two groups.

**FIGURE 1:**
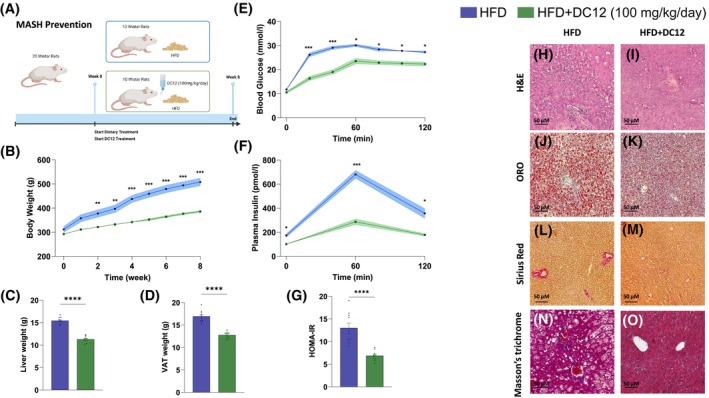
DC12 prevents obesity and MASH in DIO rodents. (A) Design of the prevention study. (B) Body weight time course. (C, D) Liver and visceral adipose tissue (VAT) weight. (E, F) Time courses of blood glucose and plasma insulin concentrations during an oral glucose tolerance test. (G) HOMA‐IR index. (H–O) Representative Hematoxylin and Eosin (H&E). (H, I) Oil Red O (ORO). (J, K) Picro Sirius Red. (L, M) Masson's Trichrome. (N, O) staining of liver sections from rats fed HFD with or without DC12 supplementation. Data are reported as mean value ± SEM of *n* = 10 animals per group. Statistical significances were calculated by unpaired two‐tailed *t*‐test and one‐way Anova with Bonferroni's correction for multiple comparisons, where appropriate. ****< 0.0001; ***< 0.0003; **< 0.003; *< 0.02.

Prevention Study (Figure 1A): Twenty Rats were fed a high‐fat diet (20% carbohydrate, 20% protein, 60% saturated triglycerides with 5% Cholesterol) (Mucedola, Milan, IT) for 8 weeks in the presence (*n* = 10 rats) or absence (*n* = 10 rats) of Dodecanedioic acid, high‐purity sodium salt (Metabolyte®) (100 mg/kg/day) dissolved in water.

Dodecanedioic acid, high‐purity sodium salt was kindly provided by Jemyll Ltd.

**FIGURE 2:**
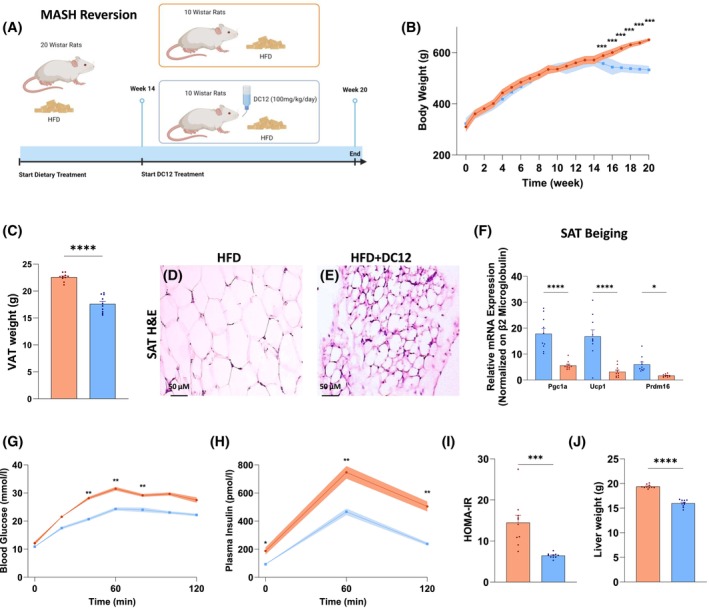
DC12 promotes weight loss and insulin sensitivity. (A) Design of the MASH reversion study. (B) Body weight time course. (C) Visceral adipose tissue (VAT) weight. (D, E) Representative Hematoxylin and Eosin (H&E) staining of subcutaneous adipose tissue (SAT). (F) Gene expression of SAT beiging‐associated gene expression, namely PPARG coactivator 1 alpha (Pgc1a), Uncoupling protein 1 (Ucp1), and PR/SET domain 16 (Prdm16). (G, H) Time courses of blood glucose and plasma insulin concentrations during an oral glucose tolerance test. (I) HOMA‐IR index. (J) Liver weight. Data are reported as mean ± SEM of *n* = 10 animals per group. Statistical significances were calculated by unpaired two‐tailed *t*‐test and one‐way Anova with Bonferroni's correction for multiple comparisons, where appropriate. ****< 0.0001; ***< 0.0002; **< 0.004; *< 0.04.

Reversion Study (Figure 2A): Twenty rats were fed a high‐fat diet (20% Carbohydrate, 20% Protein, 60% Fat) for 14 weeks followed by 6 weeks of a high‐fat diet in the presence (*n* = 10 rats) or absence (*n* = 10 rats) of Dodecanedioic acid, sodium salt (100 mg/kg/day) dissolved in water. Body weight and food intake were monitored weekly. All animal procedures were approved by the Catholic University of Rome Institutional Animal Care Committee.

### 2.2 Plasma dodecanedioic acid analysis

Blood samples were collected in heparinized tubes simultaneously from the portal and tail veins at the time of the animal sacrifice to measure plasmatic concentrations of DC12. Water was provided ad libitum the night before the animals were sacrificed. The average water consumption over 24 h was 13.5 ± 2.1 mL per animal. Plasma extraction of DC12 and its analysis by high‐performance liquid chromatography were reported elsewhere.^12^

### 2.3 Oral glucose tolerance test

All animals underwent an oral glucose tolerance test (OGTT) at the end of the study. After an overnight fasting, all rats received a 50% D‐glucose solution (1 g/kg body weight) by oral gavage. Blood samples were taken by tail bleeding and collected in EDTA tubes. All blood samples were immediately centrifuged, and plasma was divided into appropriate subsamples and stored at −20°C for further analysis. Blood glucose was measured at 0, 20, 40, 60, 80, 100, and 120 min, while plasma insulin was measured at 0, 60, and 120 min. Blood glucose levels were measured by a glucometer (Accu‐Chek, Roche Diagnostics Division, Grenzacherstrasse, CH). Plasma insulin was measured by ELISA (EMD Millipore Corporation, Billerica, MA), with a sensitivity of 0.1 ng/mL and an intra‐ and inter‐assay precision of 1.9% and 7.6%, respectively. Plasma citrate was assessed by the Citrate Assay Kit (Abcam, Cambridge, UK), with a sensitivity >0.002 mM.

### 2.4 Histology

The day of the sacrifice, fresh portions of liver and subcutaneous adipose tissue (SAT) were embedded in cryo‐embedding media (OCT) and snap frozen in liquid nitrogen. Biopsies were cut using a cryostat (5 μm) and slides stored at −20°C until analyses. Hematoxylin and Eosin staining was performed to assess hepatic steatosis. Slides were fixed in 95% ethanol, stained with hematoxylin, washed with distilled water, stained with eosin, and cleared in two changes of pure ethanol and two changes of xylene. Oil Red O was performed to assess intracellular lipid accumulation. Slides were fixed overnight with 4% formalin, stained with Oil Red O solution, and counterstain was performed with H&H solution. Sirius Red was used to detect hepatic fibrosis. Slides were fixed with 4% formalin, stained in Direct red 80, washed in acidified water, and dehydrate in 3 changes of absolute ethanol. After brief clearing in xylene, the slides were mounted in a resinous medium. Masson's trichrome staining was used as an additional method to detect hepatic fibrosis. Slides were stained using the Trichrome Stain (Masson) Kit according to the manufacturer's instructions. Images were taken with an optical microscope (Leica DM2000, Wetzlar, DE). All reagents for histological analysis were obtained from Sigma‐Aldrich (St. Louis, MO).

### 2.5 Quantitative real‐time PCR analysis

Total RNA from rats liver, SAT, and rat primary hepatocytes was extracted using the RNeasy Plus Mini Kit (QiagenGmbH, Hilden, DE) following manufacturer's instructions. A small aliquot of total RNA (3 μL) was subjected to qualitative and quantitative control using microdrop (ThermoFischer Scientific, Waltham, MA) and the assessment of the individual samples was performed using a dedicated software. Total RNA was reverse transcribed into cDNA by using iScript RT (Bio‐Rad Laboratories, Hercules, CA). SYBR Green gene expression assays were performed according to the manufacturer's instructions using the iQ™SYBR® Green Supermix (Bio‐Rad Laboratories, Hercules, CA) and the CFX96 Touch Real‐Time PCR Detection System (Bio‐Rad Laboratories, Hercules, CA). The following pairs of primer were used: Phosphoenolpyruvate carboxykinase (Pck) (forward 5′ ATGACAACTGCTGGTTGGCT 3′ and reverse 5′ CCACCACGT AGGGTGAA TCC 3′), Glucose 6‐phosphatase (G6Pc) (forward 5′ ACAGGTCCAGGAAGTCCATCT 3′ and reverse 5′ GCATGCCACCAATTACTCCAAG 3′), Glucokinase (Gck) (forward 5′ AGTTGTTGACTCTGGGCACC 3′ and reverse 5′ TTCATGTGCCCGTTGTGAGT 3′), Pyruvate kinase (Pk) (forward 5′ CTTCCCCTTGCTCTACCGTG 3′ and reverse 5′ ACCACGGAGCTTTCCACTTTC 3′), ATP‐citrate lyase (Acly) (forward 5′ ATTGGGGCTTACCTTGTCCG 3′ and reverse 5′ CCACGGTTCGGGTTTCTACA 3′), Acetyl‐CoA Carboxylase (Acc1) (forward 5′ ATTGGGGCTTACCTTGTCCG 3′ and reverse 5′ CCACGGTTCGGGTTTCTACA 3′), Fatty acid synthase (Fasn) (forward 5′ GAATCCGCACAGGCTACCAA 3′ and reverse 5′ CTGGGCTTCACCATCACCAT 3′), Diglyceride acyltransferase (Dgat) (forward 5′ AGCAGGAGTAGGCCCCATAG 3′ and reverse 5′ ATTGGGGCTTACCTTGTCCG 3′), solute carrier family 13 member 5 (Slc13A5) (forward 5′ AGAGGCAGTGGTAGTCGTGT 3′ and reverse 5′ TCCCCTTTAGCCCTTGTTCC 3′), carnitine palmitoyltransferase 1A (Cpt1A) (forward 5′ AGTGCAGAGCAATAGGTCCC 3′ and reverse 5′ AAACATCCAGCCGTGGTAGG 3′), Uncoupling protein 1 (Ucp1) (forward 5′ CGCTACACTGGGACCTACAA 3′ and reverse 5′ AGGAGTCGTCCCTTTCCAC 3′), PR/SET domain 16 (Prdm16) (forward 5′ AGTGTGTAGCTGCTTCTGGG 3′ and reverse 5′ CGTCACCGTCACTTTTGGC 3′), PPARG coactivator 1 alpha (Ppargc1a) (forward 5′ GGAGTGACATAGAGTGTGCTG 3′ and reverse 5′ AAAGCTGTCTGTGTCCAGGT 3′). mRNA expression levels were normalized to β2‐microglobulin (forward 5′ AGGACTGGTCTTTCTATCTCTTGT 3′ and reverse 5′ ACCTCCATGATGCTGCTTACA 3′), and quantification of relative gene expression, presented as a percentage of the relevant baseline, was calculated using the 2‐∆CT (comparative threshold) method.

### 2.6 Western blot analysis

Liver was homogenized in RIPA buffer containing a cocktail of protease inhibitors. Homogenates were cleared by centrifugation (19 000× *g*; 30 min, 4°C). The protein content was determined using the Bradford Protein Assay (Bio‐Rad Laboratories, Hercules, CA). Protein lysates (30 μg) were separated on 10% SDS‐PAGE, transferred onto PVDF membrane, and blocked with EveryBlot Blocking Buffer (Bio‐Rad Laboratories, Hercules, CA) for 5 min. Membranes were probed overnight with CPT1A and SLC13A5. Detection and analysis were performed, respectively, with the Chemidoc XRS Image system and Image Lab 5.0 software (Bio‐Rad Laboratories, Hercules, CA). All the results were normalized with β‐Actin. CPT1A, SLC13A5, and β‐Actin antibodies were obtained from Santa Cruz Biotecnology (Dallas, TX).

### 2.7 Rat primary hepatocytes isolation

Primary rat hepatocytes were isolated using a 2‐step perfusion method. Briefly, rat livers were perfused with PBS, containing 5 mM glucose and 0.5 mM EDTA, followed by PBS, containing 5 mM glucose, 5 mM CaCl2, and 0.5 mg/mL collagenase (Merck, Darmstadt, DE). The liver was then removed and gently in agitated Dulbecco's Modified Eagle Medium (DMEM) (Merck, Darmstadt, DE), and filtered through nylon mesh (100 μm). The cells were washed and resuspended in DMEM. Equal volumes of normal hepatocyte suspension and isotonic Percoll (Merck, Darmstadt, DE) and centrifuged.

Isolated cells were grown until confluent in Dulbecco's Modified Eagle Medium (DMEM) (Merck, Darmstadt, DE) medium supplemented with 10% fetal bovine serum (FBS) (Merck, Darmstadt, DE). Primary rat hepatocytes were stimulated with palmitic acid (400 μM) with or without the addition of Dodecanedioic acid, sodium salt (100 μM), for 24 h. Cells cultured in DMEM were used as a control.

### 2.8 In vitro citrate uptake

Primary rat hepatocytes were incubated with or without Dodecanedioic acid, sodium salt (50–1000 μM), and citrate (500 μM) for 40 min. At the end of the stimulation, citrate uptake was evaluated using the Citrate Assay Kit (Sigma‐Aldrich, St. Louis, MO) following the manufacturer's instructions, and the data analyzed with the Varioskan™ LUX multimode microplate reader (Thermo Fisher scientific, Waltham, MA).

In order to compute IC50, that is, the concentration of the competitive antagonist (DC12) required to reduce the binding of the agonist (citrate) to the hepatocyte receptor by 50%, the following log‐logistic function was used:

$$\%\text{Uptake}=K_{\min}+\frac{K_{\max}-K_{\min}}{1+{\left(\frac{X}{\mathrm{IC}50}\right)}^{\gamma }}$$

where *K*_max_ = 100% uptake, *X* is the concentration of the antagonist DC12, *K*_min_ is the minimum attainable percent uptake in the presence of very large antagonist concentrations, and *γ* is the slope around the inflection point and represents the steepness of the curve.

All the observed percent uptakes have been computed with respect to the average of the observed hepatic citrate concentrations at DC12 equal to zero.

The estimates of the model parameters were obtained by means of a generalized nonlinear least squares approach with variance increasing with increasing fitted values.

### 2.9 Plasma lipidomic

Plasma lipidomic was measured by high‐resolution mass spectrometry (UHPLC‐QTOF; Agilent Technologies) with an untargeted acquisition and targeted analysis. 10 μL of plasma was deproteinized with 150 μL of cold methanol and 10 μL of internal standard and centrifuged at 14 000 rpm for 20 min.

Lipids were separated by ZORBAX Eclipse Plus C18 2.1 × 100 mm 1.8 μm column (Agilent, Santa Clara, CA). Untargeted acquisition was set in positive electrospray ionization mode. Metabolomics Profinder MassHunter software (Agilent Technologies) was used for peak identification and target data analysis of the most significant lipids. Quantitative analysis was performed using internal standards TAG(C45:0), PC(C34:0), LPC (C17:0), SM(d18:1/17:0), and CER (d18:1/17:0) (Avanti Polar Lipids, Alabaster, AL and Larodan, Solna, SE).

### 2.10 De novo lipogenesis

De novo lipogenesis was assessed by deuterated water (^2^H_2_O) techniques. Briefly, the day before the sacrifice, rats were administered an i.p. bolus injection of ^2^H_2_O (35 mL/kg). Rats then continued to receive ^2^H_2_O (6% vol/vol) in the drinking water. The day of the sacrifice, approximately 1 mL of blood was drawn using a cardiac puncture. Blood was centrifuged at 1500× *g* for 10 min to separate plasma and stored at −80°. DNL was assessed by measuring the deuterium incorporation into hepatic free palmitic acid (i.e., enrichment): briefly, about 25 mg of liver tissues were homogenized using the Precellys Evolution Homogenizer (Bertin Instruments, Frankfurt, Germany), and lipid species were extracted with a modified Folch method (Folch, Lees et al. 1957), using 900 μL of chloroform: methanol (2:1) and 200 μL of water. The Lipid phase was dry under gentle nitrogen flux and derivatized using BSTFA +1% TMCS (Merck KGaA, Darmstadt, Germania). Enrichment of palmitate was measured by gas chromatography/tandem mass spectrometry (GC 8890/MS 7000D, Agilent, Santa Clara, CA), monitoring ions with mass‐to‐charge ratios (m/z) of 313 (M + 0) and 314 (M + 1) and corrected for baseline enrichment. DNL was calculated using free hepatic palmitate enrichment, divided by 22 (i.e., the number of exchanged hydrogens), and divided by D2O enrichment.

### 2.11 Statistical analysis

Data are expressed as the mean ± SEM unless otherwise specified. Statistical significance was set at *p* < .05 (two‐tailed). Statistical significances were calculated by unpaired two‐tailed *t*‐test and one‐way Anova with Bonferroni's correction for multiple comparisons, where appropriate. Heatmaps were used as a graphical representation of plasma lipidomic. The statistical analyses were carried out by the SPSS version 26 software (SPSS Inc., Chicago, IL, USA).

## 3 RESULTS

### 3.1 Plasma concentrations of DC12

The plasma concentration of DC12 was 76.9 ± 3.1 μM in the portal vein, while it was 40.7 ± 0.9 μM in the tail vein, suggesting that 51% of DC12 was metabolized during its passage through the liver before entering systemic circulation.

### 3.2 DC12 supplementation prevents MASH and obesity development

To assess the effects of DC12 dietary intake on MASH prevention, 20 male Wistar rats were fed a HFD for 8 weeks with or without DC12 supplementation (100 mg/kg/day) in the drinking water (Figure 1A).

We found that after two weeks of dietary intervention, rats fed HFD and DC12 showed a decrease in body weight gain when compared to HFD‐fed rats with preservation of lean body mass. By the end of the study, rats that received HFD and DC12 supplementation weighed on average 32% less than HFD‐fed rats (Figure 1B). Our results are in line with what was observed by Goetzman et al.^13^ in mice, although both the study protocol and diet were different.

In addition, the DC12 group showed a significant reduction in the weight of both liver and visceral adipose tissue (Figure 1C,D). Increased visceral adipose tissue mass is often associated with impaired glucose tolerance and metabolic syndrome.^14^ Consistently, rats fed HFD in association with DC12 showed significantly lower glycemic and insulinemic excursions in response to an oral glucose load as compared with HFD alone (Figure 1E,F). Moreover, the values of the homeostatic model assessment for insulin resistance (HOMA‐IR) were significantly lower in DC12 rats as compared with the HFD group (Figure 1G), suggesting a higher hepatic insulin sensitivity.

Obesity and glucose intolerance are associated with the development of MASH, which is recognized as the hepatic manifestation of the metabolic syndrome.^15^ Histological analysis of the liver for MASH hallmarks revealed that rats fed a HFD plus DC12 were protected from developing diet‐induced MASH. Indeed, the DC12 rats displayed an absence of significant steatosis with a reduction in lipid content, no hepatocyte ballooning or liver inflammation, as well of liver fibrosis as compared with HFD‐fed rats (Figure 1H–O).

### 3.3 DC12 supplementation promotes weight loss and adipose tissue beiging and reverses MASH

To assess the effects of DC12 dietary intake on weight‐loss and MASH reversion, 20 male Wistar rats were fed a HFD for 14 weeks followed by 6 weeks of HFD with or without DC12 supplementation (100 mg/kg/day) (Figure 2A).

Consistently with the previous results, rats fed HFD and DC12 showed a decrease in body weight starting from the second week of DC12 administration. By the end of the study (20 weeks), rats receiving HFD and DC12 supplementation weighed 46% less than HFD‐fed rats (Figure 2B). Furthermore, the weight of visceral adipose tissue was significantly lower in the DC12 group (Figure 2C). Visceral fat primarily contains white adipocytes, which store energy and contribute to metabolic risks when in excess. In contrast, subcutaneous adipose tissue (SAT) comprises both white and beige adipocytes. Beige adipocytes are metabolically active cells resembling brown fat due to their ability to burn energy rather than store it. This characteristic of SAT, especially through its beige adipocytes, may support higher energy expenditure, making it less harmful than visceral fat and potentially beneficial for metabolic health.^16^, ^17^, ^18^ Beige adipocytes are relatively small in size compared to typical white adipocytes. They contain numerous small lipid droplets and a higher number of mitochondria, which contribute to their enhanced thermogenic capacity.^18^

Accordingly, rats treated with DC12 displayed a reduction in SAT adipocyte size and increased expression of PPARG coactivator 1 alpha (Pgc1a), Uncoupling protein 1 (Ucp1), and PR/SET domain 16 (Prdm16), key genes involved in adipose tissue beiging (Figure 2D–F).

Accordingly, rats fed HFD and DC12 showed significantly lower glycemic and insulinemic levels in response to an oral glucose load as compared with HFD‐fed rats (Figure 2G,H). Moreover, HOMA‐IR values and liver weight, were significantly lower in DC12 rats than in HFD mates (Figure 2I,J) suggesting improvement of hepatic insulin resistance. Next, we assessed the hepatic expression of genes coding for key rate‐limiting enzymes of gluconeogenesis and glycolysis. Rats fed an HFD plus DC12 showed a lower expression of gluconeogenic genes and an increase in genes involved in glycolysis, in line with the decreased glucose excursion observed during the glucose load (Figure S1).

**FIGURE 3:**
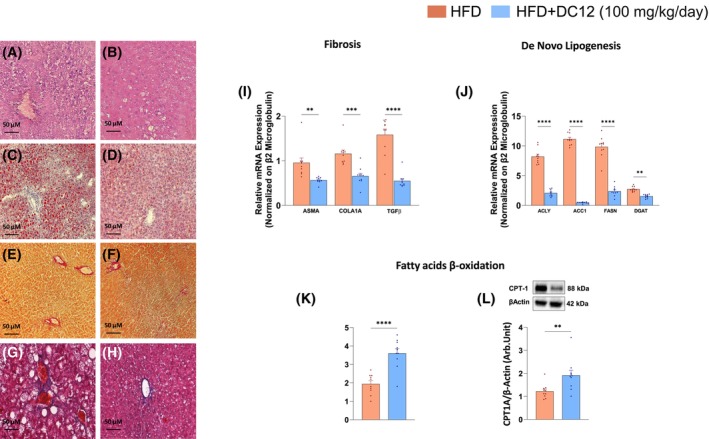
DC12 reverse MASH. (A–H) Representative Hematoxylin and Eosin (H&E) (A, B), Oil Red O (ORO) (C, D), and Picro Sirius Red (E, F), Masson's Trichrome (G, H) staining of liver sections from rats fed HFD with or without DC12 supplementation. (I) Gene expression of hepatic fibrosis marker, namely alpha smooth muscle Actin (ASMA), Collagen Type I Alpha 1 Chain (COLA1A), and transforming growth factor‐beta (TGFβ). (J) Gene expression of hepatic key rate‐limiting enzymes of de novo lipogenesis, namely ATP‐citrate lyase (ACLY), acetyl‐CoA carboxylase (ACC), fatty acid synthase (FAS), and Diacylgycerol acyltransferase (DGAT). (K, L) Gene and protein expression of Carnitine Palmitoyltransferase 1A (CPT1A), the rate‐limiting enzyme of fatty acid β‐oxidation. Data are reported as mean ± SEM of *n* = 10 animals per group. Statistical significances were calculated by unpaired two‐tailed *t*‐test. ****< 0.0001; ***< 0.0002; **< 0.002.

Histological analysis of the liver for MASH hallmarks revealed that DC12 largely improved diet‐induced MASH. Indeed, the DC12 rats displayed a significant reduction of liver steatosis and neutral lipid content as well as hepatocyte ballooning and inflammation when compared with HFD alone (Figure 3A–D). Although fibrosis did not completely reverse, we observed a substantial reduction assessed by both Sirius red and Masson's trichrome (Figure 3E–H). Indeed, the expression of hepatic fibrosis markers was significantly reduced in DC12 rats as compared with HFD‐fed rats (Figure 3I).

MASH is often associated with higher rates of DNL, which plays an important role in hepatic lipid deposition.^19^ In line with the reduction of hepatic steatosis showed by histological analysis, we observed a decrease in the four key rate‐limiting enzymes of DNL, namely ATP‐citrate lyase (ACLY), acetyl‐CoA carboxylase 1 (ACC1), fatty acid synthase (FASN), and Diacylglycerol acyltransferase (DGAT), and an increase in fatty acids β‐oxidation (Carnitine palmitoyltransferase 1 (CPT1)) in rats with MASH fed DC12 (Figure 3J–L). Moreover, hepatic DNL flux, measured by ^2^H incorporation into fatty acids following deuterated water (^2^H_2_O) administration, was decreased with DC12 (1.12 ± 0.75 vs. 5.39 ± 0.17%; *p* = .009).

### 3.4 Plasma lipidomic

People with MAFLD often display hypertriglyceridemia^20^, ^21^, ^22^ that directly correlates with the production of very low‐density lipoproteins (VLDL).^23^ Furthermore, an elevation in phosphatidylcholine (C32:0 and C32:1) was observed in patients with MASH as compared with patients with simple steatosis.^24^, ^25^

**FIGURE 4:**
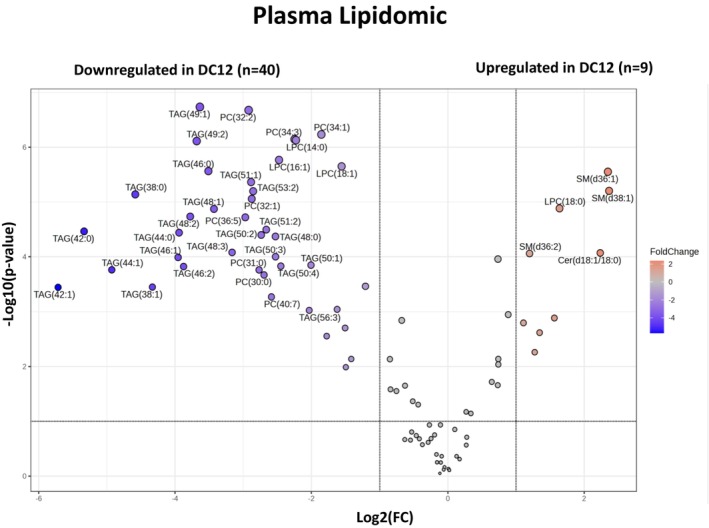
Plasma lipidomic. Volcano plot showing the most significant lipid species found by univariate analysis. Gray values indicate those lipid species that are not significantly changed (*p* > .05). Negative values (in violet) indicate downregulated lipid species, while positive (in orange) values reflect upregulated lipids in rats fed HFD and DC12 (*p* < .05).

Accordingly, plasma triglycerides, lysophosphatidylcholines and other phosphatidylcholines were significantly lower in rats fed an HFD, and DC12 than HFD alone (Figures 4 and S2). This excess fatty acid likely stimulates the synthesis and release of sphingolipids.

Indeed, we observed an increase in plasma sphingolipids (including sphingomyelins and ceramides), suggesting liver detoxification of saturated fatty acids, rather than de novo lipogenesis, which was reduced.

McGlinchey et al.^26^ found that high plasma levels of some sphingomyelins, namely SM(d36:1), SM(d36:2), and SM(d38:1), and of some phospholipids, in particular LPC(18:0), are associated with absence or minimal liver fibrosis in MASH. Accordingly, we found a similar pattern in the plasma lipidomic of rodents with MASH that received DC12, which showed a substantial reversal of liver fibrosis (Figures 4 and S2A–E). The above‐mentioned lipids were proved to be essential in predicting accurately the degree of liver fibrosis or its absence.^26^

### 3.5 DC12 reduces citrate uptake through mINDY inhibition

In addition to being a precursor of lipid and cholesterol biosynthesis, citrate also serves as an important link between glucose and lipid metabolism. mINDY mediates the transport of citrate but also of dicarboxylic acids and plays an important role in controlling cytosolic citrate concentrations.^11^, ^27^

**FIGURE 5:**
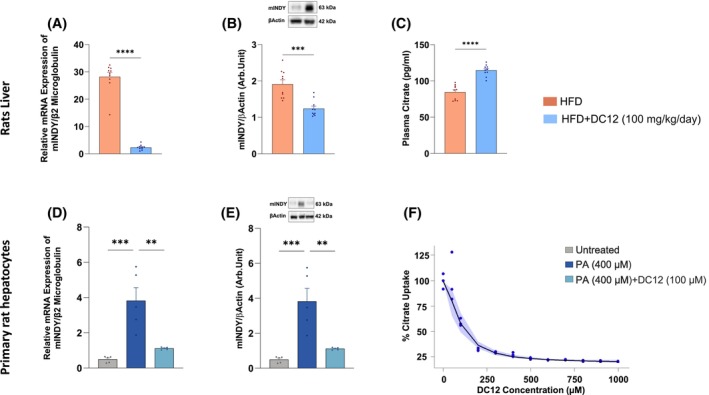
DC12 reduces citrate uptake through mINDY inhibition. (A, B) Hepatic gene and protein expression of mINDY in rats fed HFD with or without DC12 supplementation. (C) Plasma citrate concentration in rats fed HFD with or without DC12 supplementation. (D, E) Gene and protein expression of mINDY in human primary hepatocytes treated with palmitic acid (400 μM) in the presence or absence of DC12 (100 μM). (F) In vitro citrate uptake using increasing concentrations of DC12 (50–1000 μM); citrate uptake fit in the presence of increasing concentrations of DC12 with a 90% confidence band (shaded area). The following are the estimates of the parameters of the model: *K*_min_ = 19.85 ± 0.70% (CI: 18.48–21.22%); IC50 = 95.76 ± 10.13 μM (CI:75.90–115.61%); gamma = 1.75 ± 0.21# (CI: 1.33–2.17). Data are reported as mean value ± SE of *n* = 10 animals per group. Statistical significances were calculated by unpaired two‐tailed *t*‐test and Kruskal–Wallis test where appropriate. In vitro data are reported as mean value ± SE of five independent experiments (D, E) and raw data (with 95% CI in blue) of three independent experiments (F). ****< 0.0001; ***< 0.0003; **< 0.002.

To determine if the reduction in DNL was related to a decrease in citrate uptake, we measured mINDY expression both in vivo and in vitro. Rats with MASH fed a HFD plus DC12 displayed a significant reduction of mINDY expression both at gene and protein levels (Figure 5A,B). In line with the decreased expression of mINDY, we also observed an increase in plasma citrate levels in these rats, suggesting a reduction of cytosolic citrate uptake (Figure 5C).

Next, we assessed the expression of mINDY in rat primary hepatocyte cultures treated with palmitic acid (400 μM) in the presence or absence of DC12 (100 μM). In vitro stimulation with DC12 decreased gene and protein expression of mINDY (Figure 5D,E).

To confirm that DC12 could be a potential inhibitor of mINDY, we measured citrate uptake in rat primary hepatocytes using increasing concentrations of DC12 (50–1000 μM). As shown in Figure 5F, DC12 inhibited citrate transport in a dose‐dependent manner, with an estimated half maximal inhibitory concentration (IC50) value of 96 μM, meaning that DC12 displaces citrate from its transporter with a 1:1 molar concentration.

The average concentration of DC12 in the portal vein was 78.3 μM, which falls within the range sufficient to inhibit citrate transport in hepatocytes. Notably, half of the DC12 consumed with water was taken up by the liver during its first‐pass metabolism.

## 4 DISCUSSION

In this study we demonstrated that DC12 not only prevents the development of diet‐induced MASH but also promotes regression of MASH when it is already established.

Goetzman et al.^13^ have recently shown that supplementation of a 60% ultra‐HFD containing DC12 significantly reduced liver triglyceride content. Here, we demonstrate that DC12 prevents the development of MASH and reverses liver steatosis, hepatocyte ballooning, and hepatic inflammation, effectively reversing MASH. Additionally, it significantly ameliorates liver fibrosis. Liver fibrosis is associated with increased risk of new‐onset cardiovascular disease (CVD), specifically heart failure, CVD hospitalization, and all‐cause mortality.^28^

Moreover, liver fibrosis can eventually evolve to cirrhosis and to hepatocellular carcinoma.^29^ Therefore, it is the outmost clinical importance to prevent or at least improve MASH and liver fibrosis.

MASH is characterized by abnormal fat accumulation in the liver, with hepatic DNL representing one of the major factors contributing to the progression of this disease.^30^, ^31^, ^32^ The cytoplasmic citrate pool is a crucial precursor for fatty acid synthesis in the DNL pathway.^33^, ^34^ This citrate pool is replenished by two main sources: the influx via the cell membrane citrate/Na^+^ cotransporter and the efflux from the mitochondrial Krebs' cycle.

In our study we found that in vivo and in vitro administration of DC12 significantly reduced mINDY gene and protein expression and inhibited hepatic citrate uptake.

We found that during the hepatic first pass, half of the DC12 ingested with water was taken up by the liver. The concentration of DC12 in the portal vein falls within the range identified in vitro as sufficient to inhibit citrate transport in hepatocytes.

mINDY is significantly increased in the livers of human and mice with MAFLD^35^ and has been suggested as a therapeutic target with the potential to reduce both de novo lipogenesis and hepatic glucose production.^36^ Deleting this transporter in mice results in significant metabolic changes and protection against the harmful effects of a high‐fat diet.^11^, ^37^, ^38^

In the cytosol, citrate is converted into fatty acids through reactions catalyzed by ACLY, ACC, and FASN. Bempedoic acid, which is a dicarboxylic acid used to treat hypercholesterolemia, is an inhibitor of ACLY that is in the pipeline of MASH treatment.^39^ Several pan‐ACC inhibitors have recently entered clinical testing in humans, but their development was discontinued due to the onset of hypertriglyceridemia.^40^ However, some more selective ACC inhibitor drugs have progressed to phase II clinical trials.^39^

In the current study, we showed that daily oral administration of DC12 reduces de novo lipogenesis and increases fatty acid oxidation, while reversing glucose intolerance and MASH, without increasing circulating triglycerides.

Consistent with these observations, several studies have shown that the inhibition of ACLY, ACC^41^, ^42^ or FASN^43^ reduces hepatic fat content and fibrosis markers in patients with MASH while activating hepatic fatty acid β‐oxidation.^44^ Moreover, increasing the activity of CPT1A in animal models halts the hepatic accumulation of triglyceride during high‐fat feeding.^45^, ^46^, ^47^

Accordingly, in our animal model, daily administration of DC12 decreased hepatic DNL flux, plasma triglycerides, and phosphatidylcholines levels. We found increased levels of some sphingomyelins, which have been shown to be protective for the liver and are high in the absence of liver fibrosis in MASH.^48^

It is well known that about 60% of patients with type 2 diabetes also have MASH,^49^ therefore, the improvement of glucose metabolism can contribute to the improvement of histological MASH.

Importantly, we found that DC12 ameliorates insulin resistance and drastically improves glycemic response to an oral glucose challenge. An activation of a thermogenic gene program in brown or beige adipocytes can increase systemic energy expenditure and ameliorate or prevent obesity‐associated metabolic disorders.^50^ Accordingly, we found that DC12 can promote adipose tissue beiging and the activation of key genes involved in thermogenesis.

One limitation of our study is the absence of energy expenditure measurements using respiratory chambers. This limitation highlights the need for future studies to incorporate calorimetric assessments to better elucidate the full thermogenic impact of DC12 treatment.

In conclusion, our studies indicate that DC12 administration protects against MASH and reverses diet‐induced MASH by inhibiting mINDY and DNL, underlying a possible role of DC12 as a therapeutic option for the prevention and treatment of MASH.

## AUTHOR CONTRIBUTIONS

Geltrude Mingrone and Giulia Angelini designed the study. Giulia Angelini and Sara Russo carried out the study. Amalia Gastaldelli, Fabrizia Carli, Patrizia Infelise and Simona Panunzi performed lipidomic and metabolomics analysis. Simona Panunzi and Alessandro Bertuzzi performed the analyses. Giulia Angelini, Geltrude Mingrone, Stefan R. Bornstein, Erminia Lembo, and Roberta Calce wrote the first draft. All authors actively contributed to the final version.

## DISCLOSURES

GA reports consulting fees from Metadeq and GHP Scientific. GM reports consulting fees from Novo Nordisk, Eli Lilly, Boehringer Ingelheim, Medtronic, Fractyl Inc., Recor Inc. She is also Scientific Advisor of Keyron Ltd., Metadeq Inc., GHP Scientific Ltd., and Jemyll Ltd. All other authors declare no competing interests.

## Supporting information


Figure S1.

## Data Availability

The data that support the findings of this study are available in the Materials and Methods, Results, and/or Supporting Information of this article.
