# Supplementary material for: Dodecanedioic acid prevents and reverses metabolic‐associated liver disease and obesity and ameliorates liver fibrosis in a rodent model of diet‐induced obesity
Source: FASEB J. 2024 Nov 26;38(22):e70202. doi: 10.1096/fj.202402108R (PMC11599784; doi:10.1096/fj.202402108R)
Supplement: Supplementary file 1 — Figure S1. [file FSB2-38-e70202-s001.pdf]

SUPPLEMENTAL FIGURE 1

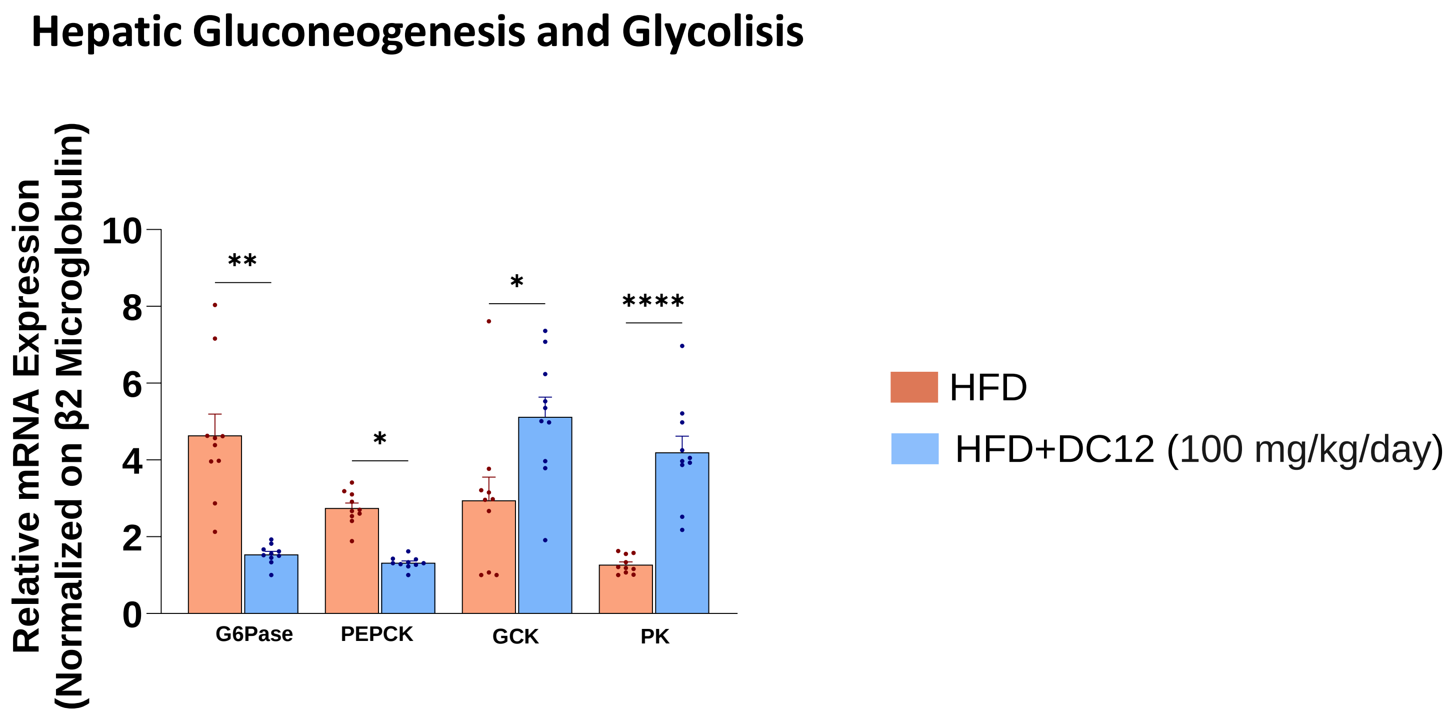

Supplementary Figure 1.

Gene expression of key limiting enzymes involved in hepatic gluconeogenesis and glycolysis, namely Phosphoenolpyruvate carboxykinase (PEPCK), Glucose 6-phosphatase (G6Pase), Glucokinase (GCK), Pyruvate kinase (PK). Data are reported as mean $\pm$ SEM of $n = 10$ animals per group. Statistical significances were calculated by unpaired two-tailed t-test.

SUPPLEMENTAL FIGURE 2

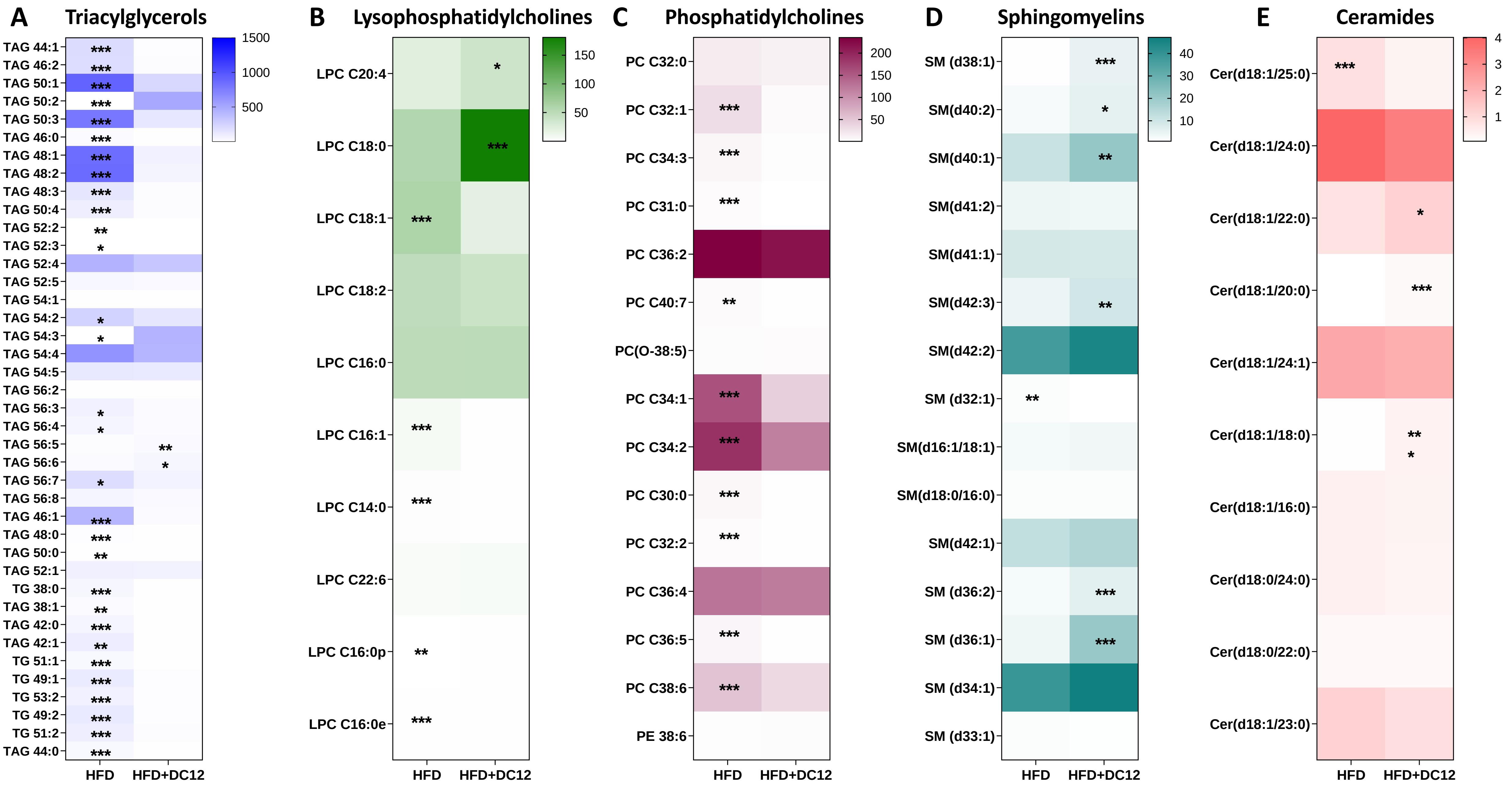

Supplementary Figure 2.

Hierarchical clustering heatmap analysis of triacylglycerols (**A**), lysophosphatidylcholines (**B**), phosphatidylcholines (**C**), sphingomyelins (**D**) and ceramides (**E**) in rats fed HFD with or without DC12 supplementation.
